# Functional inhibition of urea transporter UT-B enhances endothelial-dependent vasodilatation and lowers blood pressure via L-arginine-endothelial nitric oxide synthase-nitric oxide pathway

**DOI:** 10.1038/srep18697

**Published:** 2016-01-07

**Authors:** Yi Sun, Chi-Wai Lau, Yingli Jia, Yingjie Li, Weiling Wang, Jianhua Ran, Fei Li, Yu Huang, Hong Zhou, Baoxue Yang

**Affiliations:** 1 State Key Laboratory of Natural and Biomimetic Drugs, Key Laboratory of Molecular Cardiovascular Sciences, Ministry of Education, Department of Pharmacology, School of Basic Medical Sciences, Peking University Health Science Center, Beijing, China; 2 Institute of Vascular Medicine and Li Ka Shing Institute of Health Sciences, Chinese University of Hong Kong, Hong Kong, China; 3 Department of Anatomy and Neuroscience Center, Chongqing Medical University, Chongqing, China

## Abstract

Mammalian urea transporters (UTs), UT-A and UT-B, are best known for their role in urine concentration. UT-B is especially distributed in multiple extrarenal tissues with abundant expression in vascular endothelium, but little is known about its role in vascular function. The present study investigated the physiological significance of UT-B in regulating vasorelaxations and blood pressure. UT-B deletion in mice or treatment with UT-B inhibitor PU-14 in Wistar-Kyoto rats (WKYs) and spontaneous hypertensive rats (SHRs) reduced blood pressure. Acetylcholine-induced vasorelaxation was significantly augmented in aortas from UT-B null mice. PU-14 concentration-dependently produced endothelium-dependent relaxations in thoracic aortas and mesenteric arteries from both mice and rats and the relaxations were abolished by N^ω^-nitro-L-arginine methyl ester. Both expression and phosphorylation of endothelial nitric oxide synthase (eNOS) were up-regulated and expression of arginase I was down-regulated when UT-B was inhibited both *in vivo* and *in vitro*. PU-14 induced endothelium-dependent relaxations to a similar degree in aortas from 12 weeks old SHRs or WKYs. In summary, here we report for the first time that inhibition of UT-B plays an important role in regulating vasorelaxations and blood pressure via up-regulation of L-arginine-eNOS-NO pathway, and it may become another potential therapeutic target for the treatment of hypertension.

Urea transporters (UTs) are a family of membrane proteins that selectively transport urea driven by urea gradient across cell membrane^1^. Two mammalian UT subfamilies have been identified. UT-A subfamily has 6 members (UT-A1~UT-A6), most of which are expressed in kidney and play an important role in urine concentrating mechanism^2^. UT-B subfamily has only one member, which has a discrete tissue distribution with high expression in erythrocyte^3^, renal descending *vasa recta* (DVR)^4^, brain astrocyte^5^, testis Sertoli cell^6^, urothelial cell in bladder and ureter^7^,^8^, endothelial cell in blood vessel^9^, etc., suggesting that UT-B may have distinct function specific to its tissue localization.

The UT-B or UT-A null mouse models were generated by gene targeting strategy in order to understand their physiological significance. Phenotypic analysis of knockout mice lacking UT-B^10^ or UT-As^11^ has provided evidence for the involvement of UTs in urinary concentration. Functional deletion of UT-B or UT-A isoforms resulted in significant polyuria and a urea-selective reduction in urine concentrating ability^12^, without overt abnormalities in their main biological functions, behavior, and sensory activity. However, deletion of UT-B or UT-As did not affect glomerular filtration rate (GFR) or the clearance rate of the principal solutes (Na^+^, K^+^, Cl^−^) in urine, except for urea^12^. Therefore, these findings suggest that UTs might be useful as novel diuretic targets to excrete water without disturbing electrolyte balance.

For over half a century, diuretics have been used as the first-line antihypertensive drugs, which reduce cardiovascular and cerebrovascular events in hypertensive patients^13^. However, the long-term use of common diuretics, such as hydrochlorothiazide (HCTZ), can cause electrolyte imbalance, high blood glucose, hyperlipidemia, and hyperuricemia^14^,^15^. Therefore, UT inhibitors as diuretics used for the treatment of hypertension should have unique advantages compared with other classes of diuretics.

We have recently described that a novel UT-B inhibitor, thienoquinolin (PU-14), is a potent inhibitor of UT-B-mediated transmembrane urea transport in human, rabbit, rat and mouse with respective IC_50_ of 1.72, 1.79, 3.51 and 5.19 μmol/L^16^. PU-14 increased urine output and decreased urine osmolality without causing electrolyte disturbance or metabolic changes^16^, suggesting that PU-14 is a potential diuretic through urea-selective diuresis. In addition, PU-14 can serve as a useful tool to explore the physiological role of UT-B in animal models.

Vasodilatation is another mechanism for diuretics, such as indapamide and HCTZ, to lower blood pressure^17^. UT-B is abundantly expressed in vascular endothelial cells^9^, which might explain its wide distribution in many extrarenal tissues. Previous study showed that UT-B deletion increased nitric oxide (NO) production via L-arginine-nitric oxide synthase (NOS)-NO pathway in mouse bladder^18^, indicating that pharmacological inhibition of UT-B can affect NO production in vascular endothelial cells and vasodilatation.

This study aimed to examine the physiological role of UT-B in blood pressure regulation using UT-B null mice and UT-B selective inhibitor PU-14, and to explore the possible mechanisms. The present results suggest that UT-B is involved in regulation of vascular function and blood pressure. The main mechanism involves the up-regulation of L-arginine-endothelial nitric oxide synthase (eNOS)-NO pathway.

## Results

### UT-B deletion lowers blood pressure and augments vasodilatation in mice

**Figure 1:**
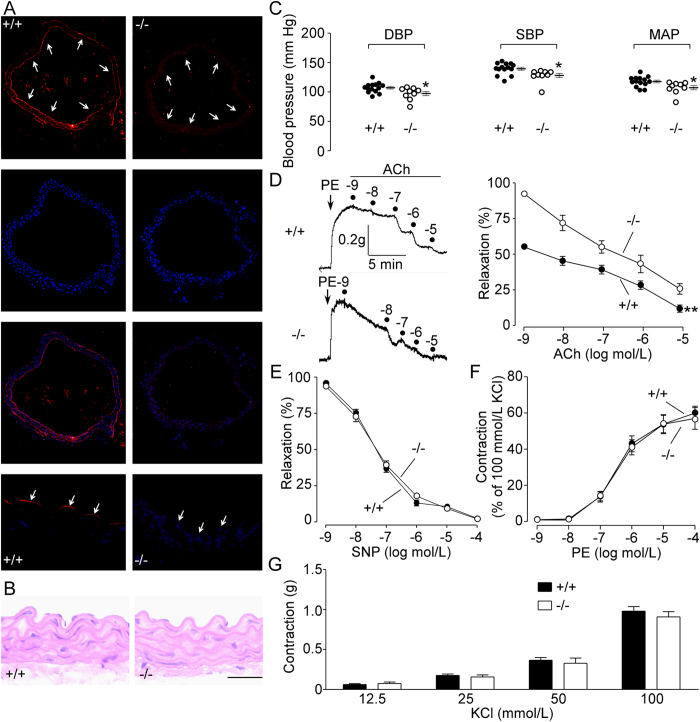
Expression and function of UT-B in mouse vascular endothelial cells. (**A**) Immunofluorescence shows UT-B (Cy3-labeled) expression in endothelium of wild-type mouse (+/+) thoracic aortas (left) but not in endothelium from UT-B null mouse (−/−) aortas (right). Cell nuclei are stained with Hoechst (blue). Arrows, endothelium. (**B**) Histology of mouse thoracic aortas with hematoxylin and eosin staining. Scale bar = 50 μm. (**C**) Diastolic blood pressure (DBP), systolic blood pressure (SBP) and mean arterial pressure (MAP) of wild-type mice and UT-B null mice. Data are mean ± SEM (n = 9–10). *P < 0.05 compared with wild-type mice (Student’s *t*-test). (**D**) Acetylcholine-induced endothelium-dependent relaxation of thoracic aortas from UT-B null mice and wild-type mice. Data are mean ± SEM (n = 10). **P < 0.01 compared with wild-type mice (ANOVA). (**E**) Endothelium-independent relaxations to sodium nitroprusside (SNP) in thoracic aortas from UT-B null mice and wild-type mice. Data are mean ± SEM (n = 10). (ANOVA). Contraction of thoracic aortas from UT-B null mice and wild-type mice induced by phenylephrine (PE, **F**) and KCl (**G**). Data are mean ± SEM (n = 7) (ANOVA).

Immunofluorescence showed that UT-B was expressed in vascular endothelial cells of thoracic aortas from wild-type mice but not from UT-B null mice (Fig. 1A). Morphological analysis with hematoxylin and eosin stain showed no structural abnormality in UT-B null mouse aortas (Fig. 1B).

Diastolic blood pressure (DBP, 97 ± 11 *vs*. 108 ± 8 mm Hg, p < 0.05), systolic blood pressure (SBP, 128 ± 11 *vs*. 140 ± 10 mm Hg, p < 0.05) and mean arterial pressure (MAP, 107 ± 11 *vs*. 118 ± 9 mm Hg, p < 0.05) were all lower in UT-B null mice than wild-type mice (Fig. 1C). UT-B null aortas pre-contracted with phenylephrine (PE, 1 μmol/L) relaxed better in response to acetylcholine (ACh) than wild-type mouse aortas (Fig. 1D). By contrast, endothelium-independent relaxations to sodium nitroprusside (SNP, 1 nmol/L–100 μmol/L) were similar in aortas from wild-type and UT-B null mice (Fig. 1E). There was no difference in contraction in response to PE (1 nmol/L–100 μmol/L) or KCl (12.5–100 mmol/L) in aortas from wild-type and UT-B null mice (Fig. 1F,G).

UT-B deficiency did not affect concentrations of main plasma electrolytes but it changed several vasoactive factors. There was no difference in plasma NO (μmol/L, 14.8 ± 3.9 *vs*. 15.2 ± 3.5), thromboxane A_2_ (TXA_2_, pg/ml, 681 ± 122 *vs*. 799 ± 141), endothelin-1(ET-1, pg/ml, 53.6 ± 1.6 *vs*. 60.0 ± 12.1) and antidiuretic hormone (ADH, ng/ml, 0.61 ± 0.13 *vs*. 0.66 ± 0.13) between wild-type and UT-B null mice (n = 10, P > 0.05). More importantly, the blood concentrations of K^+^ (mmol, 6.9 ± 0.8 *vs*. 7.0 ± 0.3), Na^+^ (mmol, 146 ± 3 *vs*. 145 ± 1.6) and Cl^−^ (mmol, 107 ± 2 *vs*. 106 ± 2) were also similar between wild-type and UT-B null mice. In addition, the plasma aldosterone remained comparable between two genotypes of mice (ng/ml, 0.22 ± 0.07 *vs.* 0.22 ± 0.05, n = 16–17, P > 0.05), further suggesting that UT-B deletion dose not disrupt electrolyte balance. However, the prostacyclin concentration was higher (ng/L, 8.0 ± 2.2 *vs*. 3.2 ± 0.9, n = 10, P < 0.01) and angiotensin II level was lower (pg/ml, 1201 ± 395 *vs*. 2291 ± 1216, n = 10, P < 0.05) in UT-B null mouse plasma compared to wild-type mouse plasma. In consistence with a previous report^10^, the present study also showed that serum urea concentration of wild-type mice was UT-B null mice was higher than wild-type mice (mmol/L, 10.2 ± 1.6 *vs*. 7.2 ± 2.2, n=10, P<0.01), while urine urea concentration was lower (mmol/L, 681.2 ± 110.0 *vs*. 1018.0 ± 106.7, n=10, P<0.01). By contrast, there was no difference in plasma creatinine concentration between wild-type and UT-B null mice (μmol/L, 29.8± 3.9 *vs*. 29.2± 4.5, n = 10, P > 0.05) despite the observation that urine creatinine concentration of UT-B null mice was lower than wild-type mice (mmol/L, 4400.0± 1227.4 *vs*. 6934.9± 1172.0, n=10, P<0.01).

### UT-B inhibitor PU-14 lowers blood pressure in rats

**Figure 2:**
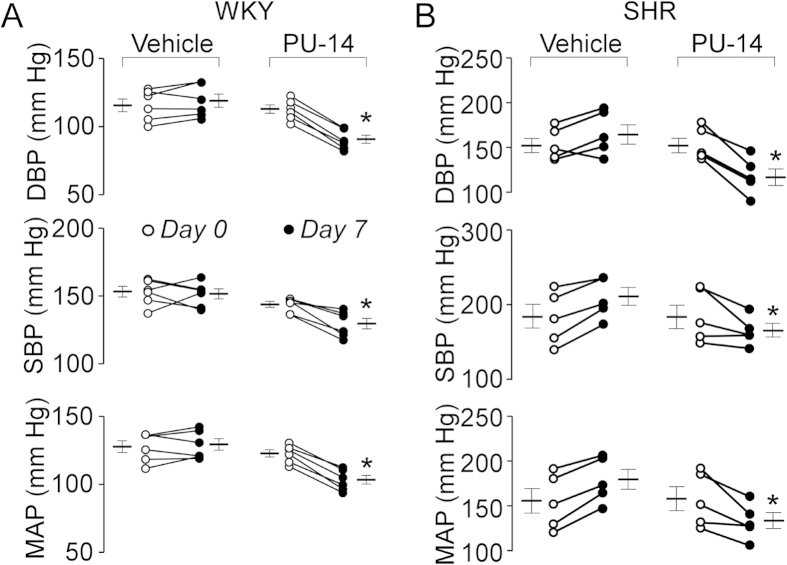
Effect of PU-14 on blood pressure in rats. Rats were subcutaneously treated with 2-hydroxypropyl-β-cyclodextrin (vehicle) or PU-14 at 50 mg/kg for 7 days. (**A**) DBP, SBP and MAP of vehicle and PU-14-treated Wistar-Kyoto rats (WKYs). (**B**) DBP, SBP and MAP of vehicle and PU-14-treated spontaneous hypertensive rats (SHRs). Data are mean ± SEM (n = 5–6). *P < 0.05 compared with day 0 of the same group (ANOVA).

To further determine the role of UT-B in blood pressure regulation, PU-14 at 50 mg/kg was subcutaneously injected every 6 hours to both Wistar-Kyoto rats (WKYs) and spontaneous hypertensive rats (SHRs). One-week treatment with PU-14 reduced DBP (91 ± 7 *vs*. 119 ± 12 mm Hg, p < 0.05), SBP (130 ± 10 *vs*. 151 ± 9 mm Hg, p < 0.05) and MAP (104 ± 8 *vs*. 130 ± 10 mm Hg, p < 0.05) in WKYs (Fig. 2 A). PU-14 treatment also lowered DBP (118 ± 21 *vs*.166 ± 25 mm Hg, p < 0.05), SBP (163 ± 19 *vs*. 210 ± 27 mm Hg, p < 0.05) and MAP (133 ± 21 *vs*. 179 ± 25 mm Hg, p < 0.05) in SHRs (Fig. 2B). There was no change in DBP, SBP and MAP in vehicle-treated WKYs or SHRs (Fig. 2A,B).

### PU-14 causes diuresis

**Figure 3:**
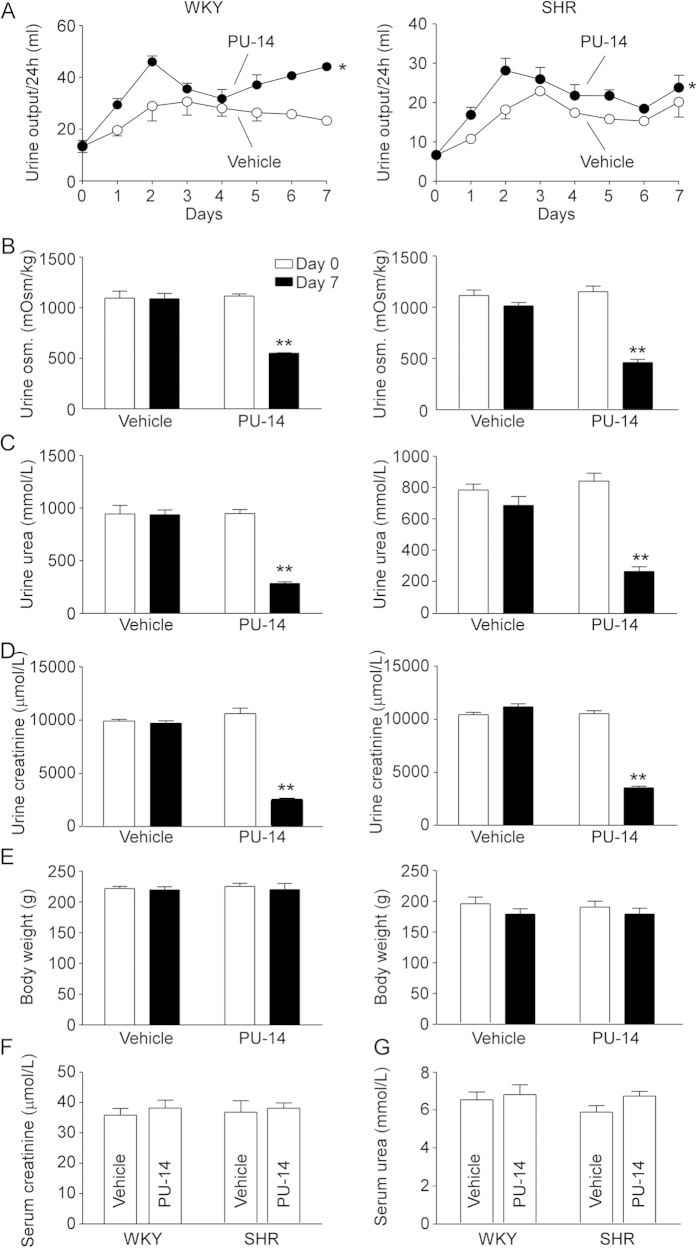
Diuretic effect of PU-14 in rats. (**A**) Urine output; (**B**) Urinary osmolality; (**C**) Urinary urea; (**D**) Urinary creatinine; (**E**) Body weight of WKYs (left) and SHRs (right). Serum creatinine (**F**) and urea (**G**) of rats. Data are mean ± SEM (n = 5–6). *P < 0.05, **P < 0.01 compared with vehicle-treated rats (General linear model with repeated measures for **A**, ANOVA for **B–G**).

PU-14 treatment increased urine output in both WKYs and SHRs (Fig. 3A). The urine osmolality, creatinine and urea concentration in PU-14-treated WKYs and SHRs were significantly lower than those in vehicle-treated rats (Fig. 3B–D). By contrast, PU-14 treatment did not change the plasma concentration of creatinine and urea in WKYs and SHRs (Fig. 3F,G). However, there was no difference in body weight between PU-14-treated WKYs and SHRs and vehicle-treated groups (Fig. 3E). In addition, the excretion of urea (mmol, vehicle-WKY, 10.2 ± 1.3; PU-14-WKY, 11.1 ± 1.3; vehicle-SHR, 9.0 ± 1.9; PU-14-SHR, 10.7 ± 2.7, n = 5–6, P > 0.05) remained similar in both PU-14-treated rats and vehicle control rats, as well as the creatinine clearance (ml/min/kg, vehicle-WKY, 11.3 ± 0.8; PU-14-WKY, 8.8 ± 2.2; vehicle-SHR, 11.1 ± 2.6; PU-14-SHR, 8.7 ± 1.2, n = 5–6, P > 0.05).

### Pharmacological inhibition of UT-B causes endothelium-dependent relaxations

**Figure 4:**
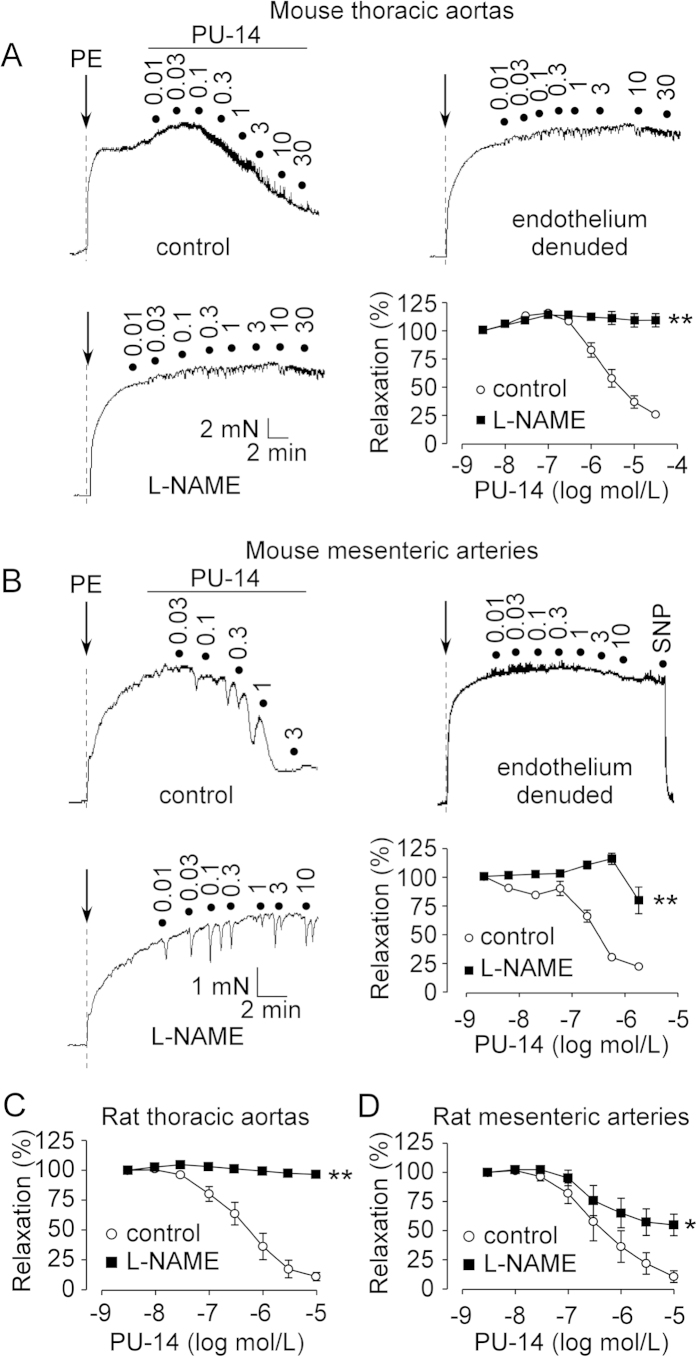
Pharmacological inhibition of UT-B induces endothelium-dependent relaxation in mice and WKYs. The relaxing effects of PU-14 in mouse thoracic aortas (**A**) and main mesenteric arteries (**B**) with endothelium and without endothelium and in L-NAME-treated endothelium-intact aortas. The relaxing effect of PU-14 in WKYs endothelium-intact thoracic aortas (**C**) and main mesenteric arteries (**D**) with and without L-NAME treatment. Data are mean ± SEM (n = 5). *P < 0.05, **P < 0.01 compared with control (ANOVA).

PU-14 produced concentration-dependent relaxations in both C57 mouse aortas (Fig. 4A) and main mesenteric arteries (Fig. 4B). The PU-14-induced relaxation was absent in endothelium-denuded rings (Fig. 4A,B). Pretreatment with 100 μmol/L N^ω^-nitro-L-arginine methyl ester (L-NAME), a nitric oxide synthase (NOS) inhibitor, blocked PU-14-induced relaxations in these arteries (Fig. 4A,B). In addition, PU-14 also produced endothelium-dependent relaxations in thoracic aortas and mesenteric arteries from WKYs and this relaxation was also inhibited by L-NAME (Fig. 4C,D). By contrast, atropine at 10 μmol/L did not affect PU-14-induced relaxation but it abolished ACh-induced relaxation (data not shown), suggesting that PU-14 is unlikely to act as the muscarinic receptor antagonistic agent.

### Inhibition of UT-B enhances eNOS-NO pathway

**Figure 5:**
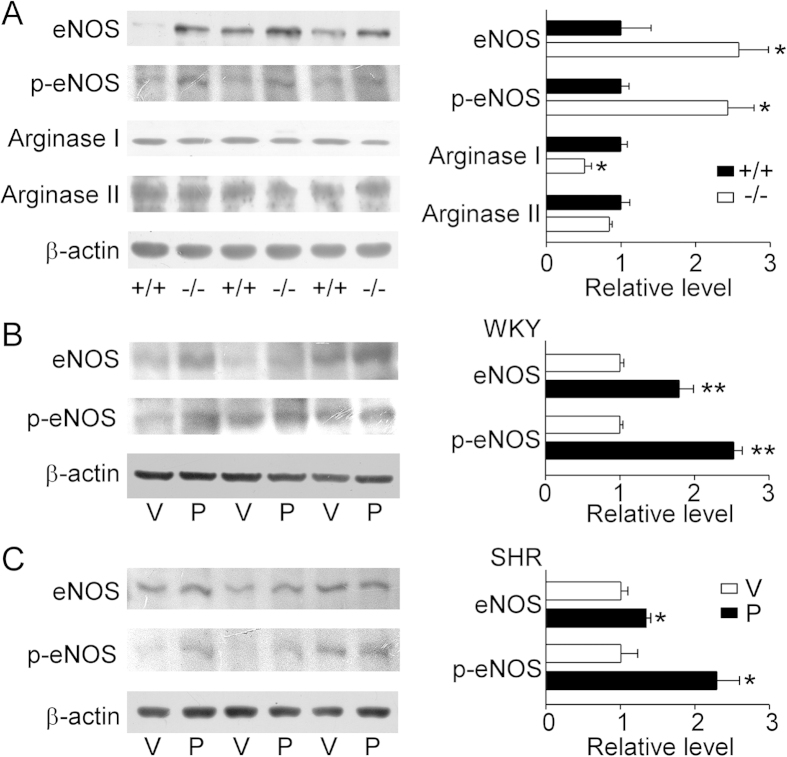
Pharmacological inhibition of UT-B enhances eNOS-NO pathway *in vivo*. (**A**) (left) The representative Western blots for eNOS, p-eNOS (ser^1177^), arginase I and arginase II protein expression in wild-type (+/+) and UT-B null (−/−) mouse thoracic aortas. (right) Bar graph shows the density ratio of eNOS, p-eNOS (ser^1177^), arginase I and arginase II to β-actin. Data are mean ± SEM (n = 3). *P < 0.05 compared with wild-type mice (ANOVA). (**B**) (left) The representative Western blots for eNOS and p-eNOS (ser^1177^) protein expression of vehicle and PU-14-treated WKYs. V: vehicle; P: PU-14. (right) Bar graph shows the density ratio of eNOS and p-eNOS (ser^1177^) to β-actin. Data are mean ± SEM (n = 3). **P < 0.01 compared with V (ANOVA). (**C**) (left) The representative Western blots for eNOS and p-eNOS (ser^1177^) protein expression of vehicle and PU-14-treated SHRs. V: vehicle; P: PU-14. (right) Bar graph shows the density ratio of eNOS and p-eNOS (ser^1177^) to β-actin. Data are mean ± SEM (n = 3). *P < 0.05 compared with V (ANOVA).

Western blot analysis of thoracic aortas showed that levels of endothelial nitric oxide synthase (eNOS) and p-eNOS (ser^1177^) were increased whereas arginase I expression was decreased in UT-B null mice, with no change of arginase II expression (Fig. 5A). In addition, the levels of eNOS and p-eNOS (ser^1177^) were elevated in aortas from PU-14-treated WKYs and SHRs with no changes in those from vehicle groups (Fig. 5B,C).

### UT-B is involved in the regulation of intracellular signaling

**Figure 6:**
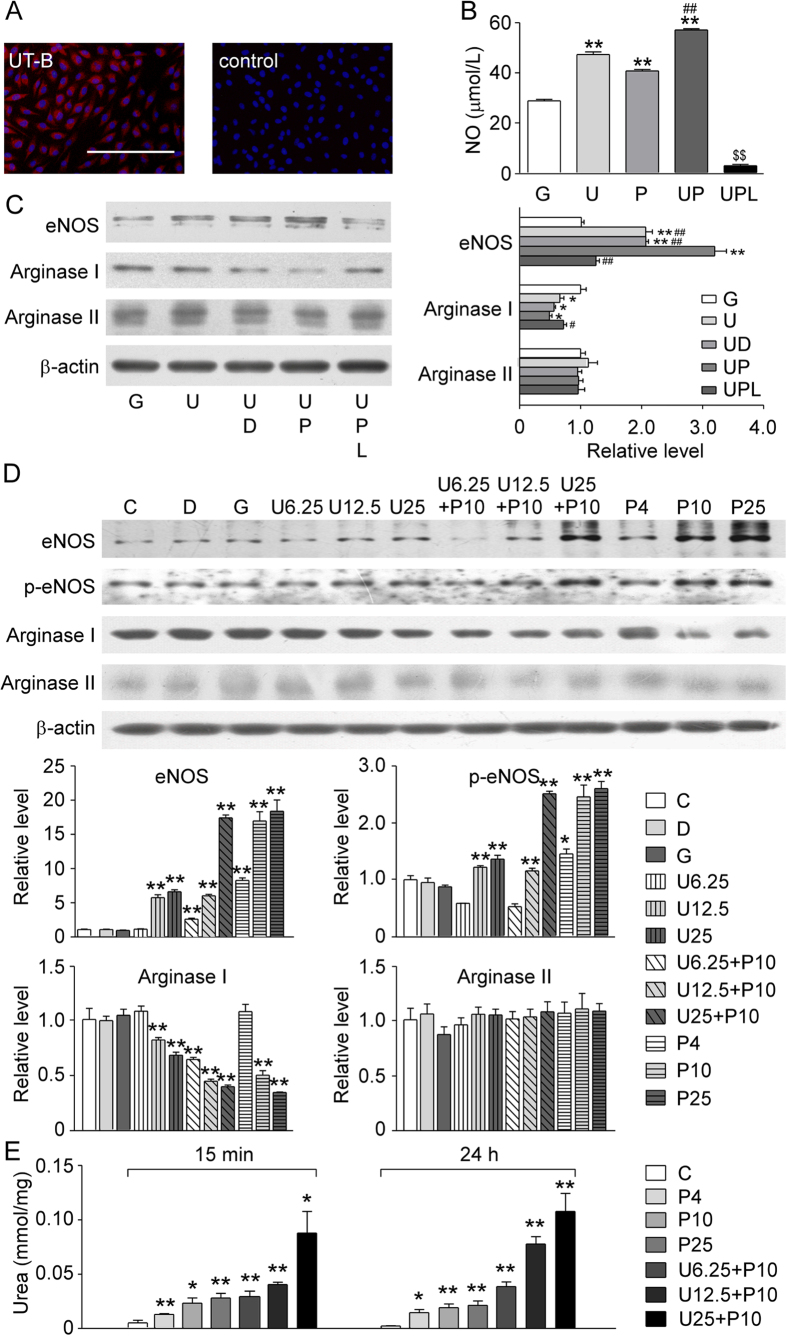
Pharmacological inhibition of UT-B regulates two metabolic pathways of L-arginine-arginase-urea and L-arginine-eNOS-NO. (**A**) Immunofluorescence of bovine aortic endothelial cells (BAECs) stained with UT-B antibody (Cy3-labeled, left) and negative control (right). Cell nuclei were stained Hoechst (blue). Scale bar = 200 μm. (**B**) NO concentration in culture medium of BAECs with following conditions. G: 25 mmol/L glucose; U: 25 mmol/L urea; P: 10 μmol/L PU-14; UP: 25 mmol/L urea + 10 μmol/L PU-14; UPL: 25 mmol/L urea + 10 μmol/L PU-14 + 100 μmol/L L-NAME. Data are mean ± SEM (n = 3). **P < 0.01 compared with G; ^##^P < 0.01 compared with U; ^$$^P < 0.01 compared with UP (ANOVA). (**C**) (left) Western blots for eNOS, arginase I and arginase II protein expression at different culture conditions. G: 25 mmol/L glucose; U: 25 mmol/L urea; U + D: 25 mmol/L urea + 0.1% DMSO; U + P: 25 mmol/L urea + 10 μmol/L PU-14; U + P + L: 25 mmol/L urea + 10 μmol/L/L PU-14 + 100 μmol/L L-NAME. (right) Bar graph shows the density ratio of eNOS, arginase I and arginase II to β-actin. Data are mean ± SEM (n = 3).*P < 0.05, **P < 0.01 compared with G; ^#^P < 0.05, ^##^P < 0.01compared with U + P (ANOVA). (**D**) (up)Western blots for eNOS, p-eNOS (ser^1177^), arginase I and arginase II protein expression at different culture conditions. C: no treatment; D: 0.1% DMSO; G: 25 mmol/L glucose; U6.25: 6.25 mmol/L urea; U12.5: 12.5 mmol/L urea; U25: 25 mmol/L urea; U6.25 + P10: 6.25 mmol/L urea + 10 μmol/L PU-14; U12.5 + P10: 12.5 mmol/L urea + 10 μmol/L PU-14; U25 + P10: 25 mmol/L urea + 10 μmol/L PU-14; P4: 4 μmol/L PU-14; P10: 10 μmol/L PU-14; P25: 25 μmol/L PU-14. (down) Bar graph shows the density ratio of eNOS, p-eNOS (ser^1177^), arginase I and arginase II to β-actin. Data are mean ± SEM (n = 3). *P < 0.05, **P < 0.01 compared with C (ANOVA). (**E**) Intracellular urea concentration. C, no treatment; P4, P10, P25: 4, 10, 25 μmol/L PU-14; U6.25 + P10, U12.5 + P10, U25 + P10: conncentration gridient urea (6.25, 12.5, 25 mmol/L) with PU-14 (10 μmol/L). Data are mean ± SEM (n = 3). *P < 0.05, **P < 0.01 compared with C at the same time (ANOVA).

Immunofluorescence showed that UT-B is expressed in bovine aortic endothelial cells (BAECs, Fig. 6A) and PU-14 showed no cytotoxicity at concentration <50 μmol/L (data not shown). Urea at <300 mmol/L did not affect cell viability^18^. To determine whether urea has any impact on NO production *in vitro*, BAECs were exposed to 25 mmol/L urea for 3 hours^9^,^19^, using 25 mmol/L glucose as an equal osmolality control. Urea incubation significantly increased NO concentration in culture medium (Fig. 6B). PU-14 (10 μmol/L) significantly increased NO production, and co-incubation of urea and PU-14 produced a greater amount of NO than urea or PU-14 alone (Fig. 6B). The NO-increasing effect of urea and PU-14 was inhibited by L-NAME. Urea in the absence and presence of PU-14 elevated the expression of eNOS and reduced the expression of arginase I (Fig. 6C).

To confirm whether the increase of intracellular urea inhibits the expression of arginase, which in turn up-regulates eNOS, endothelial cells were treated with three concentrations of urea (6.25, 12.5 and 25 mmol/L). Urea concentration-dependently decreased the expression of arginase I but not arginase II, while it increased the levels of eNOS and p-eNOS (ser^1177^) (Fig. 6D). PU-14 produced the similar effect as urea (Fig. 6D). Figure 6E shows that PU-14 increased intracellular urea levels.

### Pharmacological inhibition of UT-B induces similar vasodilatation in normal and injured vessels

**Figure 7:**
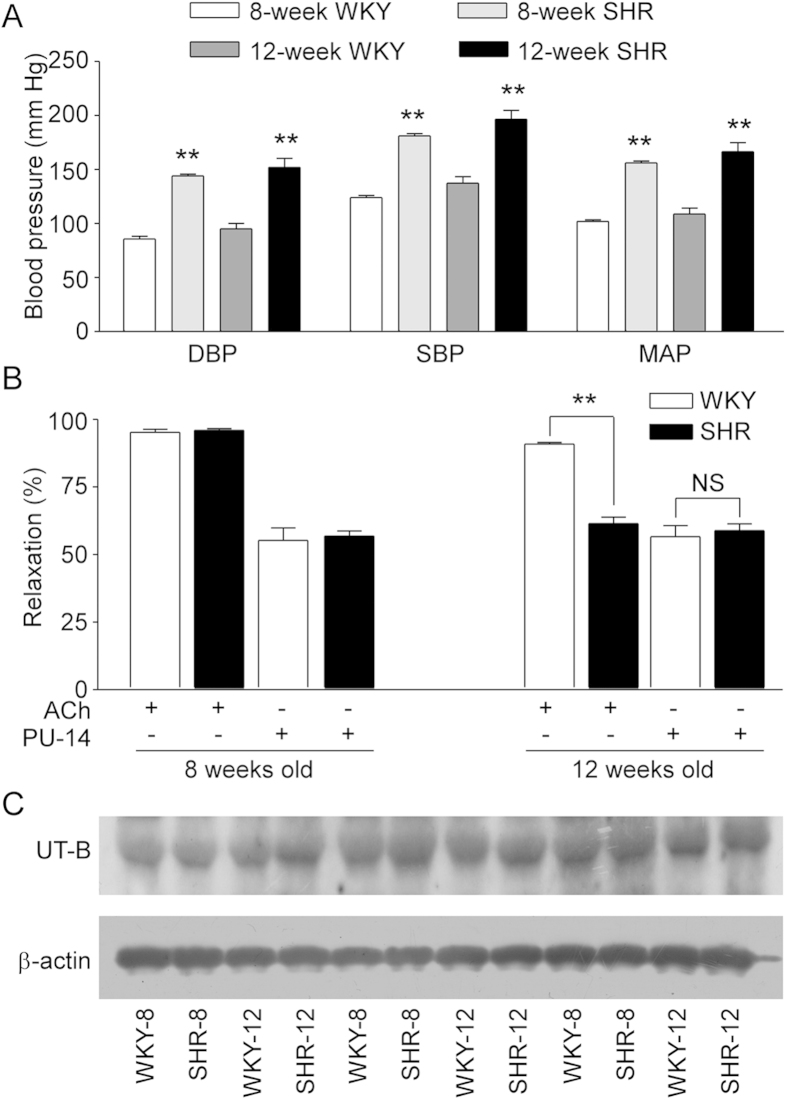
Vasodilatation effect of PU-14 in endothelial dysfunction vessels. (**A**) Blood pressure of WYKs and SHRs of 8 weeks and 12 weeks. Data are mean ± SEM (n = 8). **p < 0.01 compared with WKYs of the same age (ANOVA). (**B**) Statistics chats of endothelium-dependent relaxation to acetylcholine or PU-14 in thoracic aortas from WKYs and SHRs of 8 weeks and 12 weeks respectively. Data are mean ± SEM (n = 8). ** p < 0.01, NS: no significance compared with 12-week WKYs (ANOVA). (**C**) Western blots for UT-B in thoracic aortas of WKYs and SHRs of 8 weeks and 12 weeks. WKY-8/12, 8/12 weeks old WKY; SHR-8/12, 8/12 weeks old SHR.

Blood pressure of SHRs was higher than WKYs at the same age (Fig. 7A), although ACh-induced relaxations were similar in thoracic aortas from 8-week or 12-week old WYKs and 8-week old SHRs (Fig. 7B, 8-week WKYs, 95.2 ± 3.6%; 12-week WKYs, 90.8 ± 2.4%; 8-week SHRs, 96.0 ± 2.1%), suggesting that these arteries maintain normal endothelial function. However, ACh-induced relaxation was blunted in aortas from 12 weeks old SHRs compared with aortas from 12 weeks old WKYs (Fig. 7B, 61.3 ± 8.0% *vs*. 90.8 ± 2.4%, P < 0.01), indicating the impaired endothelial function in 12 weeks old SHRs. On the contrary, PU-14 (10 μmol/L) induced endothelium-dependent relaxations to a similar degree in aortas from 12 weeks old SHRs or WKYs (Fig. 7B, 58.7 ± 8.0% *vs*. 56.4 ± 13.3%). Finally, there was no difference in UT-B expression in aortas from SHRs and WKYs at ages of 8 and 12 weeks (Fig. 7C).

## Discussion

The original objective of this study was to determine if pharmacological inhibition of urea transporter would lower blood pressure through diuresis. It is found that both UT-B knockout in mice and UT-B inhibitor PU-14-treated WKYs and SHRs had reduced blood pressure. Previous studies showed that the antihypertensive effects of diuretics result from a moderate reduction in extracellular fluid volume and plasma volume, as reflected by the loss of body weight^20^. The present study showed that plasma antidiuretic hormone level, an index of blood volume, is comparable between wild-type and UT-B null mice. In addition, continuous PU-14 treatment did not change body weight in rats, indicating that little blood volume loss occurs after PU-14 treatment. PU-14 exerted a weak diuretic effect in SHRs, but its antihypertensive effect was stronger after seven-day PU-14 treatment. These observations led us to propose that the diuretic effect of PU-14 may have little impact on its blood pressure-lowering effect and the antihypertensive effect of PU-14 is mainly induced by vasodilator effect, with diuresis as a minor contributor.

Many studies have suggested other mechanisms underlying actions of some classical diuretics, such as HCTZ and indapamide, clinically used as the first-line antihypertensive drugs to lower blood pressure through vasodilatation^17^,^21^,^22^,^23^. The present results show that UT-B deletion and PU-14 treatment reduced blood pressure and caused relaxations of aortas and mesenteric arteries from both mice and rats. These results indicate that UT-B functional inhibition lowers blood pressure probably by vasorelaxation.

Several vasoactive substances, including NO, prostacyclin, angiotensin II, thromboxane A_2_ (TXA_2_) and endothelin-1 (ET-1) are known to participate in vascular homeostasis and blood pressure regulation. NO plays a critical role in regulation of vascular tone, control of blood flow and maintenance of vascular integrity in physiological or pathophysiological conditions^24^,^25^. As lowered blood pressure and enhanced vasorelaxation were observed in UT-B null mice, we propose that the eNOS-NO pathway may play a key role for UT-B to regulate blood pressure.

The work of Wanger *et al.* provided an important cue for us to focus on the L-arginine-eNOS-NO axis^9^,^19^. Though we failed to determine the L-arginine levels in plasma, vascular tissue or BAECs by HPLC, the present results suggest that UT-B functional inhibition decreased blood pressure by activating L-arginine-eNOS-NO pathway. First, ACh-induced relaxations were augmented in aortas of UT-B null mice compared with wild-type mice. Second, PU-14-induced relaxations depended on the presence of endothelium and the relaxations were blocked by NOS inhibitor L-NAME, suggesting that endothelium-derived NO mediates vasorelaxation caused by UT-B inhibition. The activity of eNOS controls NO production in blood vessels^26^. We observed that the expression and phosphorylation levels of eNOS were increased in aortas from UT-B null mice and PU-14-treated WKYs and SHRs. Although we do not know the detailed mechanisms leading to eNOS phosphorylation, the present study at least provides evidence that both short-term and long-term vascular effect of UT-B inhibition is associated with increased phosphorylation and expression of eNOS in arteries.

We next attempted to explore how UT-B deletion or functional inhibition may activate eNOS-NO pathway. Treatment of endothelial cells with urea (6.25, 12.5, 25 mmol/L) plus PU-14 (10 μmol/L) to mimic the condition in UT-B null mice with high plasma urea level. We found that intracellular urea accumulation increased with the treatment of increasing concentration of PU-14, accompanied by the increased levels of eNOS and p-eNOS and down-regulation of arginase I.

As UT-B inhibition caused intracellular urea accumulation, there might exist a debate whether urea can be toxic in endothelial cells. Our observation revealed that plasma urea of UT-B null mice and PU-14 treated rats were both much lower than uremia (25 mmol/L)^19^, and urea did not induce obvious cytotoxicity in BAECs under 50 mmol/L (data not shown). Moreover, previous reports indicate that the accumulation of ions and toxic substances in body fluids causes uremic symptoms. And even a prolonged increase in the concentration of urea does not produce toxic reactions, at least in patients with normal kidney function^27^.

**Figure 8:**
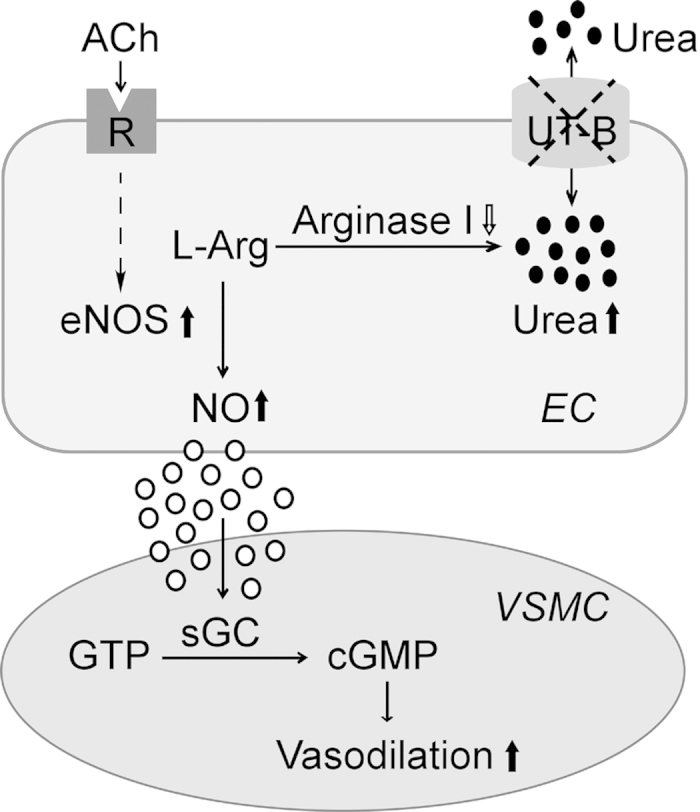
The proposed mechanism for the effect of UT-B functional inhibition on endothelial function and blood pressure. Physiologically, L-arginine is catalyzed by both arginase and eNOS at the same time, to generate urea and NO respectively. Urea is excreted though UT-B. When UT-B is defected or functionally inhibited, intracellular urea concentration is increased, which further inhibits arginase catalyzing L-arginine to urea, and elevates expression and activity of eNOS to produce more NO. The elevated bioavailability of NO mediates endothelium-dependent vasodilatation, which is likely to lower blood pressure. L-Arg, L-arginine; sGC, soluble guanylate cyclase; GTP, guanosinetriphosphate; cGMP, cyclic guanosine monophosphate; EC, endothelial cell; VSMC, vascular smooth muscle cell.

In vascular endothelial cells, two metabolic products are derived from L-arginine. One is NO catalyzed via eNOS^28^, and the other is urea catalyzed via arginase^29^. Previous studies demonstrated a beneficial effect of acute and chronic L-arginine supplementation to augment endothelium-derived NO production and thus endothelial function^30^,^31^. L-arginine lowers blood pressure in experimental hypertension^32^,^33^. Arginase is a critical regulator of NO formation by competing with NOS for their common substrate L-arginine^34^. The most direct evidence for arginase to regulate NO synthesis is that endothelial cells with stable overexpression of arginase I or arginase II decrease intracellular arginine by 25% or 11%, and reduce NO synthesis by 60% or 47%, respectively^29^. Thus, less arginase consumes less L-arginine and makes more L-arginine available for NO generation. However, an “L-arginine paradox” makes L-arginine transport, metabolism and NO synthesis even more complex. Under physiological conditions, the intracellular L-arginine level far exceeds the K_m_ of NO synthase for L-arginine, but supplement of exogenous L-arginine still increases NO production^35^,^36^. Although there are explanations for this paradox, no definite mechanism has been defined. Our study focuses more on the competitive relationship between these two metabolic pathways involving L-arginine. When UT-B is defected or functionally inhibited, the intracellular urea is accumulated to exert a feedback inhibition of arginase and to elevate the activity and expression of eNOS. As the affinity of L-arginine for eNOS is considerably greater than that for arginases^37^,^38^, increased activity and expression of eNOS induces elevated NO production (Fig. 8). However, confirmation of this hypothesis, especially the relationship between UT-B and these two competitive metabolism pathways of L-arginine, needs further study.

However, there was only an increasing trend of plasma NO level in UT-B null mice. It was reported that the plasma nitrate level might not be a reliable estimate of endogenous NO synthesis in vascular endothelial cells, because of unmeasurable confounding impact of nitrate derived from exogenous diet or nitrate-containing drugs^39^,^40^,^41^. Although NO is the primary endothelium-derived relaxing factor, other factors may be also involved in the regulation of vascular reactivity in smaller blood vessels and blood pressure^42^. We observed increase in plasma prostacyclin and decrease in plasma angiotensin II in UT-B null mice. As it has been demonstrated that cyclooxygenase-prostacyclin pathway made contribution to vasodilatation^43^,^44^, we speculate that UT-B might involve in the regulation of this pathway for unknown mechanism to make vessels relax besides eNOS-NO pathway. And other studies show close relationship between angiotensin II and blood pressure for complex factors^45^. Therefore, the present study cannot discount the contribution of these factors to beneficial effects of UT-B inhibition on endothelial function for unclear reasons^46^.

Endothelial dysfunction and reduced NO bioactivity represent prominent pathophysiological abnormalities associated with hypertensive disease^47^, though the underlying mechanism is complex. Previous studies demonstrated that endothelium- or NO-dependent vasorelaxation induced by ACh is blunted in adult SHR (12 weeks old) with developed hypertension^48^. We also found that aortas from 12 weeks old SHRs showed weaker relaxing response to ACh than that from 12-week WKYs, suggesting impaired endothelial function in 12-week SHRs. But PU-14 induced similar endothelium-dependent relaxations of aortas from both 12 weeks old SHRs and WKYs, which was consistent with the increased expression and phosphorylation of eNOS in aortas from SHRs and WKYs treated with PU-14.

In summary, the present study provides novel evidence that UT-B plays an important role in regulating vascular function and blood pressure. UT-B inhibition causes intracellular urea accumulation in endothelial cells, which decreases the arginase expression and thus increases eNOS activity and expression to produce more NO, leading to endothelium-dependent vasorelaxations. Taken together, UT-B may be a novel target for the treatment of hypertension, and UT-B inhibitors could be developed as potential antihypertensive agents.

## Materials and Methods

### Animals

Male UT-B null mice at age of 8 weeks, with a C57BL/6J genetic background were generated as described previously^10^. Male Wistar-Kyoto rats (WKYs) and spontaneously hypertensive rats (SHRs) at 8 weeks and 12 weeks old were supplied by Peking University Health Science Center (PUHSC) Laboratory Animal Service Center. All animals were housed at room temperature (23 ± 1 °C) and relative humidity (50%) under a regular light/dark cycle with free access to food and water. All animal experiments were conformed to the Guide for the Care and Use of Laboratory Animals published by the US National Institutes of Health (NIH Publication, eighth Edition, 2011) and was approved by the PUHSC Animal Experimentation Ethics Committee (Laboratory animal use license No. XYSK (JING) 2011-0039, Laboratory animal production license No. SCXK (JING) 2011-0012).

### Effect of PU-14 on WKYs and SHRs

Adult WKYs and SHRs were adapted in metabolic cages (Harvard Apparatus, Holliston, MA, USA) for 3 days before a seven-day treatment. After rat bladder was emptied by gentle abdominal massage, urine was collected by spontaneous voiding every 24 hours. PU-14 (PU-14 was home synthesized with purity at 99% as determined by HPLC) at 50 mg/kg in 40% (g/ml) 2-hydroxypropyl-β-cyclodextrin was subcutaneously injected every 6 hours (0:30 a.m., 6:30 a.m., 12:30 p.m., and 6:30 p.m.) for one week^16^. 40% 2-hydroxypropyl-β-cyclodextrin was used as a vehicle control. Two hours after the last administration, blood sample and thoracic aorta were collected under anesthesia with pentobarbital (1%) at 40 mg/kg body weight. The adequacy of anaesthesia was monitored based on the disappearance of the pedal with drawal reflex response to foot pinch. Urinary volume was measured by gravimetry, assuming a density of 1 g/ml. Urinary osmolality was measured by freezing point depression (Micro-osmometer, FISKER ASSOCIATES, Norwood, MA). Urea concentration was measured with QuantiChrom Urea Assay kit (Roche Diagnostics, Indianapolis, IN, USA).

### Hematoxylin and eosin staining and immunofluorescence

Thoracic aortas were fixed with formalin and embedded in paraffin and 6-μm paraffin sections were cut and stained with hematoxylin and eosin. Sections of the thoracic aortas from wild-type and UT-B null mice or bovine aortic endothelial cells were blocked with 2% (w/v) goat serum in 0.1 mol/L PBS, and then incubated with primary antibody (anti-UT-B, 1:200, a kindly gift from Dr. Trinh-Trang-Tan, INSERM, Paris, France) for overnight at 4 °C. After rinsing in 0.01 mol/L PBS, sections were incubated with secondary antibody (Cy3-goat-anti-rabbit, 1:200, Jackson ImmunoResearch Inc., West Grove, PA, USA), and placed in 0.1 mol/L PBS containing Hoechst (1:1000, Leagene, Beijing, China) for 1 minute to stain nuclei. The images were captured under fluorescence microscope (Leica, Wetzlar, Hesse-Darmstadt, Germany).

### Blood pressure measurement

Blood pressure was measured using a computerized tail cuff system (Kent Scientific Corporation, Torrington, CT, USA) with a photoelectric sensor. The animals were trained for 7 days before starting the measurement to prevent stress and were prewarmed to 30–32 °C with a far infrared warming pad (DCT-25, Kent Scientific Corporation, Torrington, CT, USA). Blood pressure was recorded daily at 8:30 to 10:30 a.m. and averaged from five consecutive recordings.

### Vasoreactivity measurement

Vasoreactivity assay was performed^49^,^50^. Animals were sacrificed by CO_2_ gas inhalation and bled rapidly by cutting the carotid arteries. The thoracic aortas and main mesenteric arteries were carefully isolated and placed in cold Kreb’s solution. Kreb’s solution contained (in mmol/L): 119 NaCl, 4.7 KCl, 2.5 CaCl_2_, 1 MgCl_2_, 25 NaHCO_3_, 1.2 KH_2_PO_4_ and 11 D-glucose^51^. Each artery was cut into 3–5 mm rings. Rings were held in place by means of two stiff tungsten wires (diameters, 30 μm) that were carefully passed through the lumen and fastened to clamps attached to a force transducer (Grass Instrument Co., Quincy, MA, USA) and to a micromanipulator in wire myograph. Each ring was allowed to stabilize for 60 minutes before the start of each experiment. KCl (12.5, 25, 50, 100 mmol/L) and phenylephrine (PE, Sigma, 1 nmol/L-100 μmol/L) were used to measure vessel contractility. The rings were pre-constricted with PE (1 μmol/L), and the relaxation was measured in response to cumulative concentrations of acetylcholine (ACh, Sigma, 1 nmol/L-10 μmol/L), PU-14 (0.01–30 μmol/L) or sodium nitroprusside (SNP, Sigma, 1 nmol/L-100 μmol/L). Some arterial rings were subjected to 30 minutes exposure to L-NAME (Sigma, 100 μmol/L, nitric oxide synthase inhibitor) and then endothelium-dependent relaxations in response to cumulative additions of ACh were measured. The data were analyzed with a PowerLabData Acquisition Systemand LabChart pro software (AD Instruments, Colorado Springs, CO, USA).

### NO assay

Blood samples were centrifuged at 5,000 rpm for 15 minutes and serum was collected. BAECs were treated at 80% confluence and cell culture medium was obtained after treatment for 3 hours^9^,^19^. Nitric oxide (NO) concentration in the plasma and cell culture medium was measured by conversion of nitric oxide to nitrate and nitrite using an NO assay kit (Jiancheng Bioengineering Co., Nanjing, Jiangsu, China).

### Intracellular urea accumulation assay

BAECs at 80% confluence were given different treatment. The cell samples were collected, homogenized in 100 μl of distilled water and centrifuged at 12,000 rpm for 20 minutes at 4 °C and the supernatant was for urea measurement using QuantiChrom Urea Assay kit (Roche Diagnostics, Indianapolis, IN, USA). Results were expressed relative to protein content.

### Biochemical assay

Vasoactive factors including prostacyclin, thromboxane A_2_ (TXA_2_), endothelin-1 (ET-1), angiotensin II, antidiuretic hormone (ADH) and aldosterone in plasma were assayed by radioimmunoassay kit (Beijing Northern Instrument of Biological Technology, Beijing, China). Sodium, potassium, chloride and creatinine were measured in a clinical chemistry laboratory of Peking University Third Hospital.

### Cell culture

Bovine aortic endothelial cells (BAECs, a gift from Dr. Xian Wang, Peking University Health Science Center, Beijing, China) were cultured at 37 °C in a humidified 95% air/5% CO_2_ atmosphere in Dulbecco’s modified Eagle’s medium (Gibco, Grand Island, NY, USA) supplemented with 10% fetal bovine serum (Invitrogen, Carlsbad, CA, USA), 100 U/ml penicillin, and 100 mg/ml streptomycin. Cells used in this study were from passages 3 to 8.

### Western blot Analysis

Arteries or cells were placed into RIPA lysis buffer containing protease inhibitor cocktail (Roche Diagnostics, Indianapolis, IN, USA). Protein samples (30 μg) were separated with 7.5% SDS-PAGE and transferred to polyvinylidene difluoride membranes (Amersham Biosciences, Piscataway, NJ, USA). The membrane was blocked with blocking buffer (TBS, 0.1% Tween-20, 5% non-fat milk or 2% BSA) for 2 hours at room temperature and incubated with primary antibodies against eNOS (1:1000, Cell Signaling Technology, Danvers, MA, USA), p-eNOS (ser^1177^) (1:1000, Abcam, Cambridge, MA, USA), arginase I (1:1000, Abcam, Cambridge, MA, USA), arginase II (1:1000, Santa Cruz Biotechnology, Santa Cruz, CA, USA), UT-B (1:1000, a kindly gift from Dr. Trinh-Trang-Tan, INSERM, Paris, France), β-actin (1:5000, Santa Cruz Biotechnology, Santa Cruz, CA, USA). Goat anti-rabbit IgG horseradish peroxidase (1:10000, Abcam, Cambridge, MA, USA) or goat anti-mouse IgG horseradish peroxidase (1:5000, Santa Cruz Biotechnology, Santa Cruz, CA, USA) were incubated for 60 minutes at room temperature the next day, respectively. The blots were developed with ECL kit (Applygen Technologies Inc, Beijing, China) and finally exposed to X-ray films. Relative protein expression levels were quantified by optical density analysis (Quantity-One software, Bio Rad Gel Doc 1000, Milan, Italy) and normalized to β-actin.

### Data Analysis

Statistical analysis was performed using SPSS software. All of the quantitative data are expressed as means ± SEM. Relaxations were expressed as percentage reduction in phenylephrine-induced contraction. Statistical analysis was performed using Student’s *t*-test, one-way ANOVA followed by post hoc Bonferroni test, or General linear model with repeated measures followed by post hoc Bonferroni test. P < 0.05 was considered statistically significant.

## Additional Information

**How to cite this article**: Sun, Y. *et al.* Functional inhibition of urea transporter UT-B enhances endothelial-dependent vasodilatation and lowers blood pressure via L-arginine-endothelial nitric oxide synthase-nitric oxide pathway. *Sci. Rep.* **6**, 18697; doi: 10.1038/srep18697 (2016).
